# Characteristics of informal caregivers who provide transportation assistance to older adults

**DOI:** 10.1371/journal.pone.0184085

**Published:** 2017-09-20

**Authors:** David W. Eby, Lisa J. Molnar, Lidia P. Kostyniuk, Renée M. St. Louis, Nicole Zanier

**Affiliations:** 1 University of Michigan Transportation Research Institute, Ann Arbor, Michigan, United States of America; 2 Michigan Center for Advancing Safe Transportation throughout the Lifespan (ATLAS Center), Ann Arbor, Michigan, United States of America; University of West London, UNITED KINGDOM

## Abstract

The study aim was to gain a better understanding of the characteristics of informal caregivers who provide transportation assistance and to explore the types and frequency of this assistance. A telephone survey was administered to a representative sample of 268 informal caregivers (age 45–80) who provide transportation assistance to older adults (age 70 and older) in Michigan. Responses were analyzed overall and by the caregiver sex and care recipient age. Informal transportation caregivers were: most often women; on average 61 years old; generally college educated; employed full- or part-time jobs; relatively healthy; providing care to a parent/family member 1–4 times per week, living close to the care recipient; and providing assistance by giving rides. Less than one-half of caregivers sought information to help them provide assistance. No significant burden was reported and there were few differences by sex of the caregiver of the age group of the care recipient.

## Introduction

Transportation is important for everyone. Indeed, there is increasing recognition that driving a motor vehicle is a privilege but transportation is a basic human need [1]. Transportation enables people to conduct the activities of daily life, stay connected with their world, participate in activities that make life enjoyable, and increase their quality of life. In the United States (US) and in many other countries, transportation is frequently equated with being able to drive. However, because of age-related medical conditions and the medications used to treat them, as people age into older adulthood (age 70 and older) they are more likely to experience declines in abilities needed for safe driving [2]. Declines in perceptual, cognitive, or psychomotor skills can increase the risk of a crash as well as limit personal transportation as people self-restrict their driving to times and places in which they feel safest [3, 4].

The population of the US is aging. Projections by the US Census Bureau show that the number of Americans age 65 and older will grow from 43 million in 2012 to more than 83 million in 2050 [5]. These projections also show that people age 65 and older are expected to account for about 20.9% of the population in 2050 compared to 13.7% in 2012. Even greater growth is expected for people age 85 and older, with a projected increase from about 5.9 million in 2012 (1.9% of the population) to 18.0 million in 2050 (4.3% of the population). Thus, the US is facing a coming wave of older adults who will be depending on the motor vehicle for transportation, likely experiencing declines in driving-related skills, and wanting and expecting to have their transportation needs met if driving is limited or no longer possible.

Research shows that people who cease driving will have many years of life left—6 years for men and 10 years for women—during which time they will still have transportation needs [6]. Non-driving transportation options, such as buses, taxis, and walking, are often not viable because the options are not available, acceptable, accessible, adaptable, affordable, or physically possible for older adults with medical conditions [7, 8]. Given the issues with non-personal automobile transportation, family members and friends often provide assistance in getting older adults to the places they want and need to go, serving as informal caregivers for these older adults. For example, a study in Michigan found that 94.8% of former drivers met their transportation needs by relying on informal caregivers to drive them to destinations [8].

Several studies have documented the frequency with which informal caregivers provide transportation assistance to older adults. An analysis of a US nationally representative sample of primary informal caregivers found that "shopping and/or providing transportation" was the most frequently cited caregiver activity, with 85.3% of caregivers reporting this activity [9]. A study of 380 informal caregivers to people of all ages (not just older adults) in New York found that 61–67% reported providing help with transportation [10]. This type of assistance ranked third after shopping and housework. A study of informal caregivers by family relationship for older heart attack patients found that 93% of husband caregivers, 50% of wife caregivers, and 93% of daughter caregivers provided assistance with “driving or taking the bus” [11]. A study of more than 30,000 informal caregivers of older adults in Canada found that providing transportation was the most frequently cited type of assistance by caregivers, with 39% reporting this activity [12]. Other work has also shown that husbands and other males are more likely to arrange for transportation than wives or other females [see e.g., 13, 14].

Despite the frequency with which transportation assistance tends to be provided by informal caregivers, there is little specific information about the characteristics of these caregivers, the types of transportation assistance they provide, the frequency with which they provide it, and where they seek information and/or services to help them to provide such assistance. Information about these issues is critical for the development of programs and services for informal caregivers who are tasked with helping older adults meet their transportation needs. The goal of this research was to gain a better understanding of the characteristics of informal caregivers who provide transportation assistance and to explore the types and frequency of this assistance, based on a statewide representative survey of informal caregivers of older adults in Michigan.

## Methods

### Questionnaire design and pilot testing

Data for this study came from a telephone survey administered to a representative sample of informal caregivers (age 45–80) who provide transportation assistance to older adults (age 70 and older) in Michigan. As discussed in recent research on older drivers [15], we have chosen age 70 and older as the "older adult" age group. Survey topics were derived from results of a literature review and demographic analysis [16], and discussions with experts on older adult transportation. The project team then developed survey questions for each topic. To this end, the team reviewed questionnaires that they had developed in the past, as well as published questionnaires from other researchers to find appropriate questions to include in the present surveys. The draft questionnaire was pilot-tested with five informal caregivers using a cognitive interviewing process. That is, respondents completed the questionnaire with an investigator present and “thought out loud” as they proceeded through each question, with probing questions asked as issues arose. The questionnaire was revised based on this feedback. The final survey contained 41 questions covering the following topics: demographics, driving status and frequency, work and/or volunteering activity, health, types of transportation assistance provided, characteristics of the care recipient, caregiver help information seeking, and caregiver burden.

### Sample design

The state of Michigan was stratified into seven strata defined by the seven MDOT regions. The sampling frame was developed from the Michigan Driver History File database. Because this database contained records of people who were currently licensed, as well as those with a license that was sanctioned (revoked, restricted, etc.) or had expired within the past 7 years, or a state-issued identification card, this database included both drivers and non-drivers in approximately the same proportion as found in Michigan. Samples were selected for the survey by first filtering the database for Michigan residents who were 45–80 years of age and randomly drawing replicate samples for the survey. Because the database did not include telephone numbers, a professional survey company obtained telephone numbers from commercially available databases that matched names and home addresses to telephone numbers. All study protocols and procedures were approved by the University of Michigan Institutional Review Board, Behavioral/Health Sciences.

### Data collection

The telephone survey was conducted by trained interviewers from a professional survey company who were monitored by field supervisors to ensure a high standard of quality in the data collection process. Potential respondents were screened to ensure that they had provided transportation assistance or other unpaid care to a Michigan adult age 70 or older in the past 12 months. If respondents provided care to more than one older adult, they were asked to report on the older adult to whom they provided the most transportation assistance. The survey was administered between June, 7, 2011 and July 17, 2011. In all, 5,516 contacts were made. Of these contacts, 4,649 were ineligible for some reason (e.g., not a caregiver, did not speak English, not within age range, did not provide transportation assistance, etc). Of the remaining 867 people who were eligible to participate, 30.9% agreed to participate and completed the interview. The average interview length was 14 minutes.

### Weighting

Responses were weighted to be representative of the Michigan population of informal caregivers. The weights were based on the probability of selection from the population of persons age 45–80 in each region, the probability of being an informal caregiver, and the probability of responding. Because the probability of being informal caregiver in Michigan was not known, we estimated the proportion of informal caregivers in the population of people age 45 to 80 in the Michigan Driver History Files based on how many people in that age group were contacted and the final number of caregiver respondents in each stratum. From the final survey sample disposition, it was estimated that approximately 21% of adults age 45–80 in Michigan provide informal care to an adult age 70 or older. This proportion is supported by the finding from a national survey-based study of caregivers conducted by the National Alliance for Caregiving (NAC) and AARP that 18% of the adult population of the US is engaged in some type of caregiving [17].

### Analysis

Questionnaire data were analyzed with Statistical Analysis Software (SAS) 9.4 using tools for the analysis of survey data that account for the sample design. The survey responses for each question were tabulated for the state overall and examined by the sex of the caregiver and age group of the care recipient (age 70–84 and age 85 and older). Statistical differences in group means were compared with a t-test and proportions were compared using the Rao Scott modified chi square test (computed from the Pearson chi-square statistic and a design correction based on the design effects of the proportions). In all tables, significant differences between groups are shown in boldface.

## Results

### Demographics

In reporting results from the survey, the term informal caregiver denotes someone providing transportation assistance. A total of 268 informal caregivers completed the survey. By self-report, all had provided transportation assistance to a Michigan older adult (age 70 or older) in the past 12 months. Table 1 shows the demographics of the respondents. As shown in this table, the average age of respondents was 61, two-thirds were women, nearly all were licensed drivers, and about three-quarters were currently married. Nearly all lived in their own home or apartment; a vast majority had lived at their current residence for at least the past 5 years; and the average number of people in the household was 2.4 people. Ninety-four percent of respondents were White and 2.2 percent were African American. The percentages of people reporting these races differed somewhat from 2010 Census Bureau [18] data for ages 40–84 combined for Michigan (87.9% for White and 12.1% African American). However, our sample only included people who were informal caregivers that provided transportation assistance and so it not known how these percentages compare. Respondents varied greatly in terms of household income and education.

**Table 1:** Transportation caregiver sample demographics by sex, age of care recipient, and overall.

	Sex of Caregiver		Age of Care Recipient		Overall
	Men	Women	70–84	85 and older	
**Number of respondents**	90	178	140	126	268
**Average age, years**	61.6	60.7	60.0	62.3	61.0
**(SD)**	(9.4)	(8.0)	(9.3)	(7.1)	(8.5)
**% Female**	0.0	100.0	66.4	66.7	66.4
**% Currently licensed to drive**	98.9	99.4	100.0	99.2	99.3
**% Licensed to drive in past 5 years**	100.0	100.0	100.0	100.0	100.0
**% Married**	81.1	73.6	76.4	75.4	76.3
**% Live in own home/apartment**	94.4	96.1	95.0	96.0	95.5
**% Lived 5+ years in same location**	85.6	91.6	87.9	91.3	89.6
**Average household size**	2.5)	2.4	2.5	2.3	2.4
**(SD)**	(1.1)	(1.1)	(1.1)	(1.0)	(1.1)
**% Race**					
**Caucasian**	92.2	96.1	95.7	93.7	94.0
**African American**	4.4	1.1	0.7	4.0	2.2
**Other**	3.3	1,1	2.9	0.8	11.0
**Refused**	0.0	1.7	0.7	1.5	1.1
**Annual household income**					
**$25,000 or less**	13.3	15.2	14.3	14.3	14.6
**$25,000-$49,999**	24.4	26.4	23.6	28.6	25.8
**$50,000-$74,999**	23.3	19.1	17.9	23.0	20.5
**$75,000-$99,999**	11.1	10.7	10.7	11.1	10.8
**$100,000+**	10.0	8.4	10.7	7.1	9.0
**Don’t know/refused**	17.8	20.2	22.9	15.9	19.4
**Education**					
**< High school**	5.6	4.5	3.6	5.6	4.7
**High school**	26.7	32.0	35.0	25.4	30.7
**Some college**	33.3	37.1	33.6	39.0	35.3
**College graduate**	8.9	8.4	5.7	11.1	8.7
**Graduate school**	25.9	12.9	20.0	19.1	17.7

### Caregiver employment/volunteer work status

Table 2 shows the employment and volunteer work status of the caregivers by sex, age of care recipient, and overall. Nearly one-half of caregivers worked outside the home for pay, with a significantly higher proportion of female caregivers working than male caregivers (χ^2^_(1, N = 268)_ = 4.81, *p*<0.028). There was no significant difference in the proportion of caregivers working by the age of the care recipient. Of those who reported working outside the home for pay, 68.7% worked full-time and one-quarter worked part-time. There were no statistical differences by sex of caregiver or age of care recipient. Fifty-five percent of caregivers volunteered in the community for an average of nearly 6 hours per week. Women were significantly more likely to volunteer than men (χ^2^_(1, N = 268)_ = 7.66, *p*<0.0057) and there was no significant difference in volunteering by age group of care recipient.

**Table 2:** Transportation caregiver employment and volunteer work by sex, age of care recipient, and overall (standard error of the percent/mean).

	Sex of Caregiver		Age of Care Recipient		Overall
	Men	Women	70–84	85 and older	
**% Work outside home for pay**	N = 90	N = 178	N = 140	N = 126	N = 268
	**34.6 (6.42)**	**52.3 (4.63)**	53.4 (5.40)	40.6 (6.24)	47.0 (3.86)
**If they work outside home:**	N = 36	N = 86	N = 71	N = 50	N = 122
**Full-time**	80.3 (9.30)	65.4 (6.56)	71.4 (7.59)	68.0 (9.65)	68.7 (5.55
**Part-time**	12.3 (9.30)	29.1 (6.27)	24.6 (7.50)	24.3 (8.91)	25.4 (5.26)
**Occasional**	7.4 (1.04)	5.5 (2.87)	3.9 (1.73)	7.7 (5.66)	5.9 (2.65)
**% Volunteer in community**	N = 90	N = 178	N = 140	N = 126	N = 268
	39.4 (6.24)	61.7 (4.52)	60.6 (5.44)	50.0 (5.72)	55.0 (3.91)
**Average hours of volunteer work per week**	N = 39	N = 96	N = 74	N = 61	N = 135
	**5.0 (0.98)**	**6.1 (0.88)**	5.8 (0.84)	6.0 (1.22)	5.9 (0.73)

### Caregiver health and driving

Table 3 shows the reported health and driving of informal transportation caregivers by sex, age of care recipient, and overall. About 55% of transportation caregivers rated their health as “very good” or “excellent” and about 18% rated their health as “fair” or “poor”. In general, female caregivers rated their level of health as higher than male caregivers (χ^2^_(4, N = 268)_ = 9.72, *p*<0.0453). There was no significant difference in the self-reported health of caregivers by age of their care recipients. The vast majority of informal caregivers drove regularly (95%), with only three respondents reporting that they did not drive. Of those who reported at least some driving, more than 85% reported that they drove every day or almost every day. There was no significant difference in the frequency of driving by caregiver sex or the age of their care recipient. About 84% of caregivers did not think that they would have problems with driving in the next 5 years, with no differences in answers to this question by caregiver sex or age of care recipient.

**Table 3:** Transportation caregiver health and driving by sex, age of care recipient, and overall (standard error of the percent/mean).

	Sex of Caregiver		Age of Care Recipient		Overall
	Men	Women	70–84	85 and older	
**Overall health**	N = 90	N = 178	N = 140	N = 126	N = 268
**% Excellent**	**19.1 (5.40)**	**17.3 (3.59)**	16.4 (3.78)	19.4 (4.39)	17.8 (2.96)
**% Very good**	**26.4 (6.03)**	**41.3 (4.71)**	42.7 (5.49)	31.0 (5.33)	36.8 (3.83)
**% Good**	**30.2 (6.51)**	**26.0 (4.09)**	26.0 (4.70)	28.7 (5.07)	27.2 (3.42)
**% Fair**	**11.4 (4.60)**	**14.4 (3.25)**	10.2 (2.83)	17.1 (4.36)	13.7 (2.63)
**% Poor**	**12.5 (5.42)**	**1.0 (0.45)**	4.7 (2.68)	3.8 (2.34)	4.4 (1.77)
**Driving status**	N = 90	N = 178	N = 140	N = 126	N = 268
**Regularly**	98.1 (1.17)	93.7 (1.97)	97.1 (1.06)	93.3 (2.60)	95.0 (1.43)
**Occasionally/rarely**	1.0 (0.73	5.7 (1.92)	2.9 (1.06)	5.7 (2.51)	4.3 (1.45)
**Do not drive**	0.9 (0.91	0.7 (0.47	0.0 (0.00)	1.1 (0.77)	0.7 (0.43)
**Frequency of driving**	N = 89	N = 175	N = 139	N = 124	N = 264
**Every day/almost every day**	88.4 (4.63)	84.0 (3.15)	88.4 (3.25)	82.3 (3.98)	85.3 (4.05)
**3 or 4 days a week**	10.6 (4.57)	12.1 (2.96)	9.0 (3.10)	14.3 (3.86)	11.7 (3.86)
**1 or 2 days a week**	1.0 (0.74	3.9 (1.31)	2.6 (1.03)	3.4 (1.14)	3.0 (1.60)
**Is there a chance your driving ability could become a problem within the next 5 years?**	N = 89	N = 176	N = 140	N = 124	N = 265
**Yes**	19.0 (5.17)	12.3 (2.97)	17.9 (4.14)	10.8 (3.31)	14.3 (2.62)
**No**	78.0 (5.25)	86.3 (3.08)	79.7 (4.23)	87.8 (3.35)	83.8 (2.68)
**Don’t know**	3.0 (1.03)	1.4 (1.01)	2.4 (1.44)	1.4 (0.63)	1.9 (0.78)

### Caregiver and recipient results

Table 4 presents the reported characteristics of care recipients and the type and frequency of care provided by the caregiver. As shown in this table, about 91% of caregivers were currently providing some level of unpaid care. There were no significant differences by sex of caregiver or age group of care recipient. On average, informal caregivers provided care for slightly fewer than two people. Overall, 71% of care recipients were women and women were more likely to be providing care to women than were men (χ^2^_(1, N = 267)_ = 5.82, *p*<0.016). The overall average age of the care recipient was 84 years with no significant difference by the sex of the caregiver. The average age of the care recipients in the younger age group was 79 years and 90 years for the oldest age group.

**Table 4:** Recipients and caregiver information by transportation caregiver sex, age of care recipient, and overall (standard error of the percent/mean).

	Sex of Caregiver		Age of Care Recipient		Overall
	Men	Women	70–84	85 and older	
**Providing unpaid help:**	N = 90	N = 178	N = 140	N = 126	N = 268
**Currently**	87.0 (4.37)	92.2 (2.21)	94.2 (1.91)	87.9 (3.50)	90.6 (2.01)
**Past 12 months, not now**	13.0 (4.37)	7.8 (2.21)	5.8 (1.91)	12.1 (3.50)	9.3 (2.01)
**Average number of people you have provided care for in the past 12 months**	N = 88	N = 170	N = 134	N = 122	N = 258
	1.7 (0.10)	1.7 (0.09)	1.9 (0.11)	1.6 (0.09)	1.7 (0.07)
**% Care recipient female**	N = 89	N = 178	N = 140	N = 125	N = 267
	**57.2 (7.06)**	**77.3 (4.01)**	72.6 (5.01)	70.0 (5.26)	71.4 (3.61)
**Average age of care recipient**	N = 89	N = 177	N = 140	N = 126	N = 266
	85.1 (1.02)	83.9 (0.62)	**78.5 (0.39)**	**89.9 (0.47)**	84.2 (0.54)
**Care recipient’s relationship:**	N = 90	N = 178	N = 140	N = 126	N = 268
**Spouse**	6.9 (2.85)	6.2 (2.12)	**11.4 (3.28)**	**1.6 (0.85)**	6.4 (1.70)
**Parent**	49.6 (6.32)	54.5 (4.67)	**51.2 (5.33)**	**54.5 (5.49)**	53.0 (3.77)
**Other relative**	24.7 (6.06)	18.3 (3.84)	**12.5 (3.66)**	**28.0 (5.12)**	20.3 (3.22)
**Friend**	16.2 (5.08)	18.2 (3.45)	**20.9 (4.22)**	**14.6 (3.88)**	17.6 (2.82)
**Other**	2.6 (1.32)	2.8 (1.52)	**4.1 (2.17)**	**1.4 (0.73)**	2.7 (1.13)
**Care recipient’s marital status**	N = 81	N = 160	N = 121	N = 119	N = 241
**Married**	31.9 (7.15)	19.8 (4.05)	28.0 (5.50)	19.4 (4.66)	23.4 (3.55)
**Divorced**	2.3 (1.36)	5.1 (2.22)	6.3 (2.62)	2.4 (2.03)	4.2 (1.61)
**Widowed**	57.1 (7.53)	68.0 (4.73)	57.5 (5.98)	71.1 (5.40)	64.8 (3.99)
**Single, never been married**	8.8 (3.33)	7.1 (2.64)	8.2 (3.30)	7.1 (3.19)	7.6 (2.26)
**% Care recipient who live with caregiver**	N = 90	N = 178	N = 140	N = 126	N = 268
	17.5 (5.29)	20.1 (3.85)	19.5 (4.14)	19.3 (4.51)	19.3 (3.09)
**Care recipient lives in:**	N = 69	N = 137	N = 107	N = 98	N = 206
**Own home/apartment**	87.1 (4.76)	82.0 (4.00)	88.6 (3.85)	73.6 (4.94)	83.5 (3.12)
**Other’s home/apartment**	4.5 (4.06)	2.2 (1.40)	1.0 (0.77)	4.7 (2.98)	2.9 (1.56)
**Senior/retirement community**	0.6 (0.63)	6.8 (2.66)	5.4 (2.77)	4.6 (2.65)	5.0 (1.88)
**Assisted living community**	4.7 (1.91)	5.3 (2.53)	3.6 (2.61)	6.6 (2.78)	5.1 (1.88)
**Nursing /long-term care facility**	3.1 (1.82)	3.6 (1.55)	1.4 (0.96)	5.5 (2.12)	3.5 (1.20)
**Distance the care recipient lives from caregiver**	N = 71	N = 141	N = 110	N = 100	N = 212
**Within 20 minutes**	76.3 (6.85)	82.7 (4.11)	84.1 (4.57)	77.2 (5.38)	80.7 (3.50)
**20 minutes to 1 hour**	18.5 (6.61)	12.7 (3.63)	13.0 (4.54)	16.1 (4.81)	14.5 (3.22)
**Between 1 and 2 hours**	1.4 (0.97)	1.2 (0.74)	1.8 (1.04)	0.8 (0.60)	1.3 (0.59)
**More than 2 hours**	3.8 (1.62)	3.4 (2.12)	1.2 (0.58)	5.9 (3.04)	3.5 (1.55)
**Frequency of providing assistance to care recipient**	N = 89	N = 177	N = 138	N = 126	N = 266
**Every day /almost every day**	26.4 (6.28)	27.4 (4.12)	25.9 (4.65)	28.1 (5.09)	27.1 (3.44)
**3 or 4 times a week**	20.1 (5.89)	14.6 (2.98)	16.4 (3.90)	16.3 (3.90)	16.3 (2.72)
**1 or 2 times a week**	23.7 (5.94)	41.8 (4.57)	35.3 (5.03)	37.7 (5.61)	36.4 (3.77)
**A few times a month**	18.7 (5.79)	10.3 (2.62)	17.6 (4.12)	7.8 (2.89)	12.8 (2.56)
**Once a month or less**	11.1 (3.80)	5.8 (2.05)	4.7 (1.74)	10.1 (3.23)	7.4 (1.85)
**% of care recipients with problems in:**	N = 90	N = 178	N = 140	N = 126	N = 268
**Vision**	49.0 (7.11)	45.2 (4.77)	41.1 (5.41)	51.1 (5.67)	46.4 (3.94)
**Mobility**	59.0 (6.95)	68.5 (4.46)	65.9 (5.28)	65.5 (5.40)	65.6 (3.73)
**Memory/Cognition**	36.6 (6.70)	35.0 (4.47)	31.1 (4.77)	39.6 (5.47)	35.5 (3.71)
**Other medical condition**	33.4 (5.98)	37.1 (4.47)	**46.5 (5.39)**	**26.1 (4.72)**	36.0 (3.71)
**% of caregivers who help with:**	N = 90	N = 178	N = 140	N = 126	N = 268
**Transportation**	100 (—)	100 (—)	100 (—)	100 (—)	100 (—)
**Using the telephone**	24.6 (6.24)	27.3 (4.29)	**19.6 (4.40)**	**33.5 (5.53)**	26.5 (3.49)
**Shopping**	18.2 (5.34)	25.5 (4.19)	21.1 (4.45)	25.7 (4.93)	23.3 (3.32)
**Food preparation**	27.9 (6.02)	41.2 (4.69)	37.5 (5.34)	37.3 (5.34)	37.2 (3.77)
**Housekeeping**	46.0 (6.78)	53.9 (4.69)	51.1 (5.50)	52.4 (5.25)	51.5 (3.84)
**Laundry**	27.1 (6.28)	37.3 (4.62)	31.9 (5.11)	36.7 (5.50)	34.2 (3.72)
**Taking medications**	32.7 (6.89)	36.5 (4.63)	34.2 (5.37)	36.8 (5.46)	35.4 (3.80)
**Managing finances**	38.3 (6.70)	40.8 (4.64)	37.9 (5.42)	42.4 (5.51)	40.0 (3.82)
**Other**	9.7 (4.50)	2.8 (1.15)	6.4 (2.80)	3.4 (1.58)	4.9 (1.61)
**% of caregivers who specifically help with:**	N = 90	N = 178	N = 140	N = 126	N = 268
**Bathing /showering**	14.7 (5.12)	21.3 (3.93)	18.0 (4.31)	20.4 (4.56)	19.3 (3.13)
**Getting dressed**	9.9 (4.93)	16.8 (3.52)	12.5 (3.59)	16.4 (4.31)	14.7 (2.29)
**Going to the bathroom**	13.1 (5.12)	15.6 (3.48)	15.1 (4.10)	14.8 (4.05)	14.9 (2.84)
**Getting in/out of bed/chair**	22.0 (5.88)	24.3 (4.20)	22.5 (4.71)	24.5 (5.00)	23.6 (3.40)
**Incontinence/diapers**	13.8 (5.16)	16.1 (3.48)	15.6 (4.11)	15.0 (4.04)	15.4 (2.85)
**Feeding him or her**	11.1 (4.61)	8.2 (2.85)	8.0 (3.12)	10.2 (3.67)	9.1 (2.40)
**None of these activities**	72.6 (5.93)	66.3 (4.56)	70.1 (5.06)	66.5 (5.31)	68.1 (3.65)

Respondents reported on the relationship with the care recipient to the caregiver. The results showed that 53% of recipients were a parent of the caregiver, 6% were spouses, 20% were other relatives, and 18% were friends. The relationships between the caregivers and younger care recipient were significantly different than the relationships between caregivers and older recipients (χ^2^_(4, N = 266)_ = 14.75, *p*<0.0053). Caregivers of people age 70–84 were more likely to be spouses when compared to the older care recipient age group and less likely to be a relative other than spouse or parent. There was no significant difference in the relationship by the sex of the caregiver. In terms of marital status, nearly two-thirds of recipients were widowed and another 23% were married. The marital status of the care recipient did not significantly differ by the sex of caregiver or age group of recipient.

About 20% of the care recipients lived with the caregiver, with no differences by sex of caregiver or recipient age group. Of those who did not live with the caregiver, 84% lived in their own home and about 10% lived in assisted living or retirement communities, with no differences by sex or age group. Overall, 81% of caregivers lived within 20 minutes of the care recipient, another 15% lived within 21–59 minutes, and about 4% lived more than 2 hours away. There were no significant differences in living arrangements by caregiver sex or recipient age group.

Caregivers reported providing frequent assistance to the recipient, with 27% of caregivers providing care every day or nearly every day and 80% providing care at least 1 or 2 times per week. There were no significant differences by caregiver sex or recipient age group. Respondents reported that recipients had a wide variety of functional problems, with 46% having vision problems, 66% mobility problems, 36% cognition-related problems, and 36% other medical conditions. There were no significant differences by caregiver sex or recipient age group, with the exception that caregivers were more likely to report “other medical conditions” for care recipients age 70–84 than for those age 85 and older (χ^2^_(1, N = 266)_ = 7.97 *p*<0.0048).

Caregivers were asked to report on whether they provided assistance for a number of instrumental activities of daily living (IADLs). In addition to providing transportation assistance, the most common types of assistance were: housekeeping (52%), managing finances (40%), food preparation (37%), taking medications (35%), laundry (34%), using the telephone (27%), and shopping (23%). These trends did not differ by the sex of the caregiver or age of care recipient, except for telephone use; caregivers were more likely to help older care recipients with telephone calls (χ^2^_(1, N = 266)_ = 4.19 *p*<0.0407). Respondents were also asked about providing assistance for activities of daily living (ADLs). About 68% reported that they did not provide assistance for any ADLs. Of those who did provide ADL assistance, the percentages ranged from 9% to 24%, depending on the specific ADL. There were no significant differences by sex of caregiver or age group of the care recipient.

### Transportation assistance provided

Table 5 presents results on the driving and transportation assistance provided by the caregiver. Overall, about 59% of care recipients had their own vehicle. Vehicle ownership among older care recipients was significantly lower than for the younger care recipients (χ^2^_(1, N = 265)_ = 4.35 *p*<0.0371). Overall, only 15% of care recipients drove regularly, 14% drove occasionally, and 64% did not drive or had never driven. The older group of care recipients drove less frequently than the younger care recipients (χ^2^_(4, N = 267)_ = 8.78 *p*<0.0668). There was no statistical difference in the frequency of recipient’s driving by sex of caregiver.

**Table 5:** Driving and transportation assistance by caregiver sex, age of caregiver’s care recipient, and statewide (standard error of the percent/mean).

	By Sex of Caregiver		Age of Care Recipient		Overall
	Men	Women	70–84	85 and older	
**% care recipient with a vehicle**	N = 90	N = 177	N = 139	N = 126	N = 267
	59.1 (6.70)	58.6 (4.69)	**66.5 (5.04)**	**50.8 (5.70)**	58.7 (3.86)
**% care recipients who drive**	N = 89	N = 178	N = 139	N = 126	N = 267
**Regularly**	15.5 (5.32)	15.1 (3.46)	**21.2 (4.71)**	**9.2 (3.37)**	15.2 (2.88)
**Occasionally**	14.3 (4.90)	13.3 (3.30)	**16.4 (4.28)**	**10.6 (3.41)**	13.6 (2.72)
**Rarely**	7.5 (3.62)	7.3 (2.70)	**8.6 (3.23)**	**6.2 (2.98)**	7.4 (2.16)
**Does not drive anymore**	52.7 (7.23)	55.0 (4.76)	**43.2 (5.41)**	**65.4 (5.48)**	54.3 (3.94)
**Never drove**	10.0 (3.80)	9.4 (2.80)	**10.6 (3.34)**	**8.6 (3.19)**	9.6 (2.30)
**% who provide type of transportation assistance:**	N = 90	N = 178	N = 140	N = 126	N = 268
**Gives ride in a car**	99.6 (0.36)	96.8 (1.92)	97.8 (1.95)	97.5 (1.95)	97.6 (1.36)
**Accompany on other form of transportation**	4.9 (3.29)	2.6 (0.84)	2.5 (0.92)	4.1 (2.11)	3.3 (1.15)
**Arrange for someone else to drive**	28.6 (6.73)	22.5 (3.94)	22.8 (4.74)	25.8(5.02)	24.4 (3.41)
**% who provide transportation assistance for trips to:**	N = 90	N = 178	N = 140	N = 126	N = 268
**Medical or dental services**	91.6 (2.18)	90.3 (2.52)	87.7 (3.32)	93.5 (1.92)	90.7 (1.90)
**Shopping or errands**	58.3 (6.99)	67.8 (4.23)	58.5 (5.24)	71.4 (5.03)	64.9 (3.62)
**Social or recreational activities**	51.2 (7.05)	61.1 (4.68)	**50.1 (5.51)**	**66.5 (5.34)**	58.2 (3.90)
**Family or personal business**	56.7 (6.99)	63.6 (4.55)	**53.1 (5.57)**	**70.3 (4.88)**	61.6 (3.78)
**School or religious activities**	30.0 (6.12)	33.8 (4.52)	26.1 (4.84)	39.4 (5.61)	32.7 (3.70)
**Other purpose**	6.2 (1.91)	9.6 (2.92)	5.2 (1.86)	11.9 (3.73)	8.6 (2.14)
**Frequency of providing rides for care recipient**	N = 88	N = 169	N = 133	N = 122	N = 257
**Every day or almost every day**	11.6 (4.32)	7.3 (2.45)	8.2 (2.44)	9.0 (3.38)	8.6 (2.13)
**3 or 4 times a week**	20.4 (5.75)	18.2 (3.86)	15.4(3.97)	22.4 (4.92)	18.9 (3.17)
**1 or 2 times a week**	33.9 (7.00)	39.1 (4.84)	38.1 (5.64)	37.1 (5.64)	37.5 (3.94)
**A few times a month**	18.6(5.40)	22.5 (3.86)	25.1 (4.81)	17.1 (3.95)	21.3 (3.13)
**Once a month or less**	15.6 (5.05)	13.0 (3.36)	13.2 (3.87)	14.5 (3.99)	13.8 (2.79)

When caregivers were asked about the types of transportation assistance they provided, 98% reported that they gave rides in a car, 24% helped arrange for someone else to drive the recipient, and 3% accompanied the recipient while using some other form of transportation. There were no statistical differences by sex of caregiver or age of recipient. Caregivers reported that they provided transportation assistance for a variety of trip purposes: medical/dental (91%), shopping/errands (65%), family/personal business (62%), social or recreational activities (58%), and school/religious activities (33%). Care recipients in the older age group were significantly more likely than care recipients in the younger age group to get assistance for trips for family or personal business (χ^2^_(1, N = 266)_ = 5.52 *p*<0.0188) and to social/recreational activities (χ^2^_(1, N = 266)_ = 4.65 *p*<0.0310). There was no difference in transportation assistance by care recipients’ age group for other trip purposes, and there were no differences in transportation assistance for all trip purposes by sex of caregivers. Of those caregivers who drove recipients, most provided rides 1–2 times per week (37%), a few times per month (21%), 3–4 times per week (19%), once a month or less (14%), or every day (9%) with no significant differences by caregiver sex or recipient age group.

### Information sources to assistant in providing transportation assistance

The survey explored if, what, and where caregivers sought information and services to help them provide transportation assistance and these results are shown in Table 6. Overall, 40% of respondents sought information and services to help them with providing care, with no significant difference by age of recipient. Analysis showed that female caregivers were more likely than male caregivers to seek information, although this test was marginally significant (χ^2^_(1, N = 268)_ = 3.76 *p*<0.0526). Caregivers who indicated that they sought information/services, were also asked to report the type of information that they sought. Respondents gave a wide range of answers ranging from caregiver counseling and support (31%) to respite care (18%). There were no significant comparisons by either sex of caregiver or age of the care recipient on the type of information sought. Respondents were asked about where they went to acquire needed information or services. Again, a wide variety of information sources were reported, with doctors/health care professionals being the most frequently reported (55%). There were several significant differences between groups. Female caregivers (χ^2^_(1, N = 104)_ = 4.80 *p*<0.0285) and caregivers of younger recipients (χ^2^_(1, N = 103)_ = 4.54 *p*<0.0331) were more likely to seek information from the Internet. Caregivers of recipients in the older age group were more likely than caregivers of recipients in the younger age group to seek information from newspapers (χ^2^_(1, N = 104)_ = 3.71 *p*<0.0540).

**Table 6:** Transportation caregiver information seeking by sex, age of care recipient, and overall (standard error of the percent).

	Sex of Caregiver		Age of Care Recipient		Overall
	Men	Women	70–84	85 and older	
**% caregivers who sought information/services to help them provide assistance**	N = 90	N = 178	N = 140	N = 126	N = 268
	29.1 (6.47)	44.3 (4.67)	38.1 (5.24)	41.3 (5.63)	39.8 (3.84)
**% caregivers sought the following:**	N = 29	N = 75	N = 50	N = 53	N = 104
**Caregiver training/education**	19.4 (8.92)	32.9 (7.16)	35.7 (9.31)	25.1 (7.97)	30.0 (6.00)
**Caregiver counseling/support**	25.9 (8.85)	32.7(7.23)	39.1 (9.38)	24.4 (7.85)	31.2 (6.09)
**Respite care**	25.1 (11.91)	16.2 (5.68)	24.8 (8.80)	12.3 (5.30)	18.1 (4.98)
**Transportation services**	12.6 (4.47)	26.9 (6.90)	20.8 (7.93)	26.7 (8.36)	23.8 (5.68)
**General financial support**	34.3 (14.0)	22.3 (6.16)	22.8 (8.10)	27.0 (8.02)	24.9 (5.53)
**Other information/services**	31.2 (13.8)	28.5 (6.42)	20.6 (6.71)	36.1 (8.47)	29.1 (5.67)
**% care givers sought information from:**	N = 29	N = 75	N = 50	N = 53	N = 104
**Doctor/health professional**	55.8 (13.7)	54.1 (7.51)	49.6 (9.61)	58.5 (8.65)	54.5 (6.49)
**Other family/friends**	41.4 (13.9)	37.7 (7.30)	40.0 (9.72)	37.5 (8.81)	38.5 (6.32)
**Senior center**	20.9 (9.21)	33.6 (7.02)	25.5 (8.21)	35.9 (8.65)	30.8 (5.87)
**Community group**	24.5 (12.0)	20.8 (6.34)	17.9 (7.87)	25.2 (8.06)	21.6 (5.49)
**Government agency**	21.9 (13.5)	24.4 (6.60)	25.6 (8.61)	22.5 (7.79)	23.9 (5.74)
**Paid caregiver service**	8.7 (3.51)	18.2 (5.75)	9.6 (5.54)	22.1 (7.44)	16.1 (4.64)
**Newspaper**	5.3 (3.81)	7.4 (3.52	**1.5 (1.46)**	**12.0 (5.34)**	7.0 (2.87)
**Internet**	**7.8 (4.14)**	**27.6 (6.81)**	**35.3 (9.50)**	**12.5 (5.34)**	23.2 (5.56)
**Other sources**	3.1 (3.14)	13.0 (4.65)	9.0 (5.51)	12.6 (5.22)	10.8 (3.72)

### Caregiver burden

Finally, the survey addressed caregiver burden by having respondents complete the Bakas Caregiving Outcomes Scale (BCOS) [19]. This scale included 15 questions related to possible changes in life resulting from providing care to a recipient, such as level of energy, time for family activities, emotional well-being, and time for social activities. On each item, respondents answered on a 7-point scale, indicating the degree of change ranging from –3 “changed for the worst” to +3 “changed for the best,” with 0 indicating “did not change.” The answers for the 15 item were summed to get an overall score that could range from -45 to +45. The results (Table 7) showed that, overall, caregivers indicated very little burden associated with providing care. Male caregivers reported less burden than female caregivers, although this difference only approached being significant (t_(259)_ = -1.91, *p*<0.0570). There was no significant difference in BCOS scores between caregivers of younger and older care recipients. Using the same 7-point scale, respondents also indicated which number best described in general how their life had changed as a result of caring for the older adult. While the overall average for this item was close to zero (indicating no positive or negative change), male caregivers reported a significantly more positive life change resulting from being an informal transportation caregiver than female caregivers (t_(259)_ = -2.01, *p*<0.0453). There was little difference in the life change score of the informal caregivers by the age of their care recipients.

**Table 7:** Transportation caregiver burden by sex, age of care recipient, and statewide (standard error of the percent/mean) [95^th^ CI].

	Sex of Caregiver		Age of Care Recipient		Overall
	Men	Women	70–84	85 and older	
**Average Overall BCOS Score**	N = 90	N = 176	N = 139	N = 125	N = 266
	**7.18 (2.54**	**1.61 (1.43)**	3.44 (1.35)	3.24 (2.21	3.30 (1.29)
	[2.18–12.18]	[-1.21–4.43]	[0.79–6.09]	[-1.11–7.59]	[0.76–5.84]
**How has life changed?**	N = 90	N = 173	N = 138	N = 123	N = 263
	**0.53 (0.20)**	**0.03 (0.15)**	0.25 (0.15)	0.12 (.20)	0.18 (0.12)
	[0.13–0.92]	[-0.27–0.33]	[-0.05–0.55]	[-0.28–0.52]	[-0.06–0.43]

## Discussion

This study represents the first detailed investigation into the characteristics of informal caregivers who provide transportation assistance. The study found that informal transportation caregivers for Michigan’s older adults: were most often women; were on average 61 years old; were generally college educated; had full- or part-time jobs; were relatively healthy; were generally providing care to a parent or other family member; lived close to the care recipient; and provided care 1–4 times per week. These characteristics are similar to the caregiver demographics reported in previous larger-scale studies [e.g., 9, 10, 14, 17, 20]. For example, in a nationally representative sample of informal caregivers, Wolff and Kasper [9] found that 67% of informal caregivers were women, were providing care to a spouse or family member in nearly 80% of cases; their mean age was 62.5 years, they were generally in good or excellent health, and 89% lived within 10 minutes of the care recipient. Based on these results, it appears that informal caregivers in Michigan who provide transportation assistance are similar to other informal caregivers.

Results from this study indicated that nearly all caregivers provided transportation assistance by driving the recipient themselves. Many caregivers also reported that they helped to arrange for others to drive the recipient. These findings suggest that the personal automobile is integral for the personal mobility of older adults in Michigan, especially for those who no longer drive, consistent with results of other studies [see e.g., 8]. Very few caregivers utilized public or private non-driving transportation modes when providing transportation assistance. Further research should investigate the reasons for this finding, as the use of public and private transportation services could lessen the burden of providing transportation assistance.

Transportation assistance was provided for a variety of trip purposes. Most commonly, these purposes had to do with meeting basic needs such as medical care and shopping, but many were also for meeting secondary needs such as social, recreational, or religious activities. These results suggest that if caregivers are going to utilize non-driving modes of transportation assistance, these services will need to be flexible enough to accommodate a wide range of trip purposes.

This study found that less than one-half of caregivers sought information or services to help them provide assistance to the care recipient. This suggests that most informal transportation caregivers in Michigan are either not aware that the information and services exist or they do not know where to look for them. Caregivers cited a variety of sources from which they sought information about caregiving, with the most frequent sources being the medical profession, family/friends, and senior-oriented organizations and agencies. A small portion used the Internet and very few caregivers reported using traditional media outlets (TV, radio, or newspaper) for getting caregiving information. In addition, few male caregivers sought any information to help them provide care. Collectively, these results suggest that information and services for helping caregivers provide assistance to Michigan older adults need to be better marketed, particularly to men. Given the widespread presence of information and services for helping caregivers provide transportation and other kinds of assistance on the Internet, it would also be beneficial to encourage caregivers of older adults to use the Internet in their search for information. Several sites of national and state organizations already exist for helping caregivers provide transportation assistance (such as the *Safe Drivers Smart Options* website in Michigan) [21], yet these sites are not being widely utilized. Determining whether this is from a lack of knowledge about using the Internet, not knowing where to find the sites on the Internet, or the Internet sites not providing useful information, would be a fruitful research project.

In this study, informal caregivers in Michigan did not report being burdened by providing care. Indeed, scores on the BCOS showed slight benefits of providing care statewide and for both sexes and care recipient age groups. While earlier work on informal caregiver burden reported significant health burden [22], more recent work has not supported this conclusion (see [23] for a review). Further, in the psychometric testing of the revised BCOS among caregivers of stroke survivors, slightly positive scores were also found [24]. Thus, this study finding seems to be in agreement with contemporary studies of informal caregiver burden.

Finally, the study examined differences among caregiver responses by sex of the caregiver and age group of the care recipient. Overall, there were very few differences among these groups. This suggests that both male and female caregivers are similar in demographics and provide generally the same level and type of transportation assistance. The lack of major differences in caregiver transportation assistance by age group of the recipient supports the conclusion that it is not age per se that impacts a person's ability to drive or use other forms of transportation, but rather the heath conditions a person has and/or the medications that he or she are taking [e.g., 2, 3].

This study had several strengths including the use of a statewide representative sample of 268 informal caregivers who provided transportation assistance to an older adult in Michigan, and development of a questionnaire instrument that incorporated, to the extent possible, items used in other surveys with demonstrated reliability and validity. In addition, a broad range of topics were explored including preferences for information and services, as well as caregiver burden. Limitations included reliance on self-report and the potential bias that can be introduced with survey responses.
